# Socioeconomic Inequalities and Occupational Injury Disability in China: A Population-Based Survey

**DOI:** 10.3390/ijerph120606006

**Published:** 2015-05-28

**Authors:** Haochen Wang, Gong Chen, Zhenjie Wang, Xiaoying Zheng

**Affiliations:** Institute of Population Research, WHO Collaborating Center on Reproductive Health and Population Science, Peking University, Beijing 100871, China; E-Mails: woody_37@126.com (H.W.); chengong@pku.edu.cn (G.C.); wangzhenjie1981@aliyun.com (Z.W.)

**Keywords:** occupational injury disability, socioeconomic inequalities, China

## Abstract

*Objective*: To estimate the prevalence of occupational injury disability (OID) and to examine the socioeconomic status of OID in China. *Methods*: The data derived from the China National Sample Survey on Disability in 2006 involving people aged 16–59 years old. Descriptive statistics are used to measure OID’s prevalence, and a binary logistic regression is used to identify the risk factors. *Results*: The population-weighted prevalence of OID is 1.81 (95% confidence interval (CI): 1.67–1.94). Socioeconomic risk factors include male sex, older age, living in urban areas, junior high school education, income below the poverty line, a lack of occupational injury insurance, living in the western region and working in high-risk occupations. *Conclusions*: OID is common among Chinese people aged 16–59 years old. Being male or older and having a lower income are risk factors for OID, similar to the results of previous research, but education is different. More training and education needs to be implemented to prevent OID.

## 1. Introduction

Occupational injuries are common, and worldwide approximately 313 million work accidents occur each year. Globally, 400,000 people die each year due to work-related accidents. The burden of disease that results from injuries and related diseases accounts for 4% of global GDP annually [1]. Although occupational injuries have gradually decreased in developed countries [2], the number of work accidents has markedly increased in developing countries, especially in China [3]. Socioeconomic conditions are very relevant to occupational injuries [4]. Previous research suggested that age, education level, insurance, gender and job position were associated with occupational injuries in developed countries [5,6,7,8,9,10]. In developing countries, studies suggested that poverty, educational level and work training were related to occupational injuries [11,12,13].

In China, as a populous developing country, rapid industrialization has led to rapid economic development, but this growth has also been accompanied by occupational injury disability (OID) rates that needs attention. However, there is very limited research on OID in developing countries, especially in China. In this study, we aim to analyze socioeconomic inequalities and OID in China based on the Second National Survey of Persons with Disabilities from 2006.

## 2. Methods and Data 

### 2.1. Study Population and Sampling

The data we used are based on a nationally representative population-based data set of the second China National Sample Survey on Disability from 2006 to investigate the prevalence, causes, and severity of disabilities as well as individuals’ living conditions and health services needs. This survey was approved by the State Council, the Leading Group of the China National Sample Survey on Disability, and was conducted by the National Bureau of Statistics. A strategy of multistage, stratified random cluster sampling with probability proportional to size was adopted to select 2,526,145 non-institutionalized civilians in mainland China [14]. A four-stage sampling strategy was adopted in each provincial stage with four administrative units (*i.e.*, county, town, village and community). A total of 734 counties, 5964 communities and 771,797 families were chosen for the survey. In April and May 2006, the survey was carried out across China. This sampling scheme was reviewed by experts from the Division of Statistics of the United Nations [15]. It is representative of the Chinese total population [16]. 

### 2.2. Interviewing Procedures

More than 20,000 trained interviewers and 6000 doctors, accompanied by 50,000 assistants who were familiar with the conditions in specific communities, visited each chosen family to collect data using a standardized questionnaire to screen for likely disabled individuals [16]. The questionnaire was used to inquire about hearing disability, physical disability and intellectual disability (Table 1) [14]. All of the participants who responded “yes” to any of the corresponding questions were subsequently examined by the designated physicians for further disability screening and confirmation. These physicians examined the participants following the diagnostic manual to provide the final diagnosis and to assess the severity of the disability. Ultimately, 78,137 people among all of the respondents were confirmed as disabled. The survey also collected data on socioeconomic parameters, including age, gender, residence, marital status, employment status, education, family size and family income [17]. After the survey, a reinvestigation of 99 communities was carried out to check the data’s quality. The reinvestigation indicated that only 1.31 per 1000 of the overall residents and 1.12 per 1000 of persons with a disability were not initially recorded [18]. During the survey, medical and rehabilitation suggestions were provided to those individuals diagnosed as disabled. This study was approved by the State Council of China, and informed consent was obtained from each participant. If the interviewers believed that a participant could not meet the condition for giving consent (e.g., <18 years old), the respondents’ legal guardian would give consent.

**Table 1:** Definitions of different types of disability and corresponding questions in the survey.

Types of Disability	Definition	Questions in the Survey
Hearing	Hearing disability refers to permanent hearing loss of varying degrees from any cause or the inability to hear at all or to hear clearly any nearby sound or voice. These deficits affect daily life and social activities.	Do you or your family members have hearing problems?
Physical	Physical disability refers to a loss of motor function of varying degrees or to limitations in movements or activities resulting from deformed limbs or body paralysis (palsy) or from deformity caused by damage to the structure or function of those body parts involved in mobility.	Do you or your family members have any difficulty walking, standing, squatting, climbing the stairs, grasping, washing and rinsing, or dressing?
Intellectual	Intellectual disability refers to lower than normal intellectual ability and is accompanied by adaptive behaviour disorders. This kind of disability results from impairment of the structure and functions of the nervous system, limits individual activity and participation, and requires all-round, extensive, limited, or intermittent support.	Do you or your family members have any difficulty studying?

### 2.3. Occupational Injury Disability

Occupational injury refers to workers were injured by harmful factors or developed occupational diseases during their working or work-related activities, according to the Trial Procedures for Industrial Injury Insurance for Enterprise Employees, issued by the government in 1996, after the interviewers screened for disability using the questionnaire (Table 1), the potentially disabled cases would be examined by physicians. If the causes of disability were approved as occupational injury according to the definition, the physicians would record the disabled individuals as a person with an OID. The International Statistical Classification of Diseases 10th Revision (ICD-10) was used to confirm the diagnosis of OID, including hearing disability, limb disability and intellectual disability [19]. WHO-DASII was the grading tool to assess the severity of the OID, which was graded as mild, moderate, severe, or extremely severe, among those people with OID (ICF) [20]. The classification systems, screening methods, grading standards, diagnostic tools and scales on disabilities in this survey were validated in two pilot studies [21]. 

### 2.4. Socio-Economic Variables

In this study, OID was defined as a binary dependent variable, *i.e.*, yes or no. The individuals who had an OID were marked as 1, and the individuals who were not disabled were marked as 0. This classification means that the comparison group included people who were not disabled. Education level was classified as illiteracy, elementary school, junior high school, or senior high school and above. In the Labor Law of the People’s Republic of China, the working-age of the population ranges from 16–59 years old. Therefore, according to the law, individuals in the 16–59 age range with OID were studied in this report. Among all of the disabled people in the survey, 2989 were caused by occupational injury, and these individuals comprised the study group. The distribution of disabilities in these 2989 people was 77.0% (2302) limb disability, 21.3% (637) hearing disability and 1.7% (50) intellectual disability. The age group was defined as 16–29, 30–39, 40–49, or 50–59 years old. We also defined gender as man or woman, residence as rural or urban, marital status as single, married, divorced or widowed, ethnicity as Han or minority Han, possession of occupational injury insurance as binary (*i.e.*, yes or no), average annual family income as higher or lower than the national poverty line, and the geographical location as eastern, central or western region. The occupational classification included office workers, research managers or technical personnel, technical personnel in finance, business services, administrative staff, agriculture-related workers, heavy industry workers, light industry and assembling workers, timber workers and production operators [16].

### 2.5. Statistical Analysis 

We used standard weighting procedures to calculate the inverse probability of inclusion for an individual survey respondent in the multistage sampling frame to construct the sample weights while taking into account the complex survey sample design [22]. Population-weighted numbers and weighted proportions were calculated where appropriate. Logistic regression was used to calculate the adjusted odds ratios (ORs) and 95% confidence intervals (CIs) for OID. Statistical significance was set at a two-tailed *p* value of <0.05. The statistical analyses were performed using SAS ver. 9.2 (SAS Institute, Inc., Cary, NC, USA).

## 3. Results and Discussion

### 3.1. Results

#### 3.1.1. Prevalence of OID

Table 2 indicates the socio-demographic patterns of persons aged 16–59 years old with OID in China, based on the 2006 survey. The weighted number of people aged 16–59 was 847.7 million, and the number of people with an OID was approximately 1.5 million. The prevalence was 1.81 per 1000 (95% CI: 1.67–1.94). The majority of individuals with OID were male, lived in a rural area, were under the poverty line and had a low education level. Moreover, the prevalence of OID was significantly lower among individuals with insurance than among individuals without insurance.

**Table 2:** Distribution of demographic characteristics among persons with OID in China, aged 16–59, 2006.

Demographic Characteristic	Weighted Number and Proportion	Weighted Prevalence (per 1000) and 95% CI
Total	1,532,775 (100%)	1.81 (1.67–1.94)
Age		
16–29	96,952 (6.3%)	0.40 (0.32–0.49)
30–39	286,408 (18.7%)	1.25 (1.11–1.39)
40–49	481,291 (31.4%)	2.40 (2.23–2.56)
50–59	668,876 (43.6%)	3.87 (3.69–4.05)
Gender		
Female	265,188 (17.3%)	0.64 (0.51–0.78)
Male	1,266,938 (82.7%)	2.98 (2.85–3.12)
Ethnicity		
Minority	121,271 (7.9%)	1.54 (1.44–1.65)
Han	1,411,013 (92.1%)	1.85 (1.75–1.96)
Residence		
Rural	1,000,365 (65.3%)	1.75 (1.58–1.93)
Urban	532,016 (34.7%)	1.94 (1.76–2.11)
Education		
Senior high school or above	116,267 (7.6%)	1.54 (1.41–1.67)
Junior high school	548,634 (35.8%)	2.44 (2.27–2.62)
Elementary	658,101 (42.9%)	1.92 (1.75–2.09)
Illiteracy	209,552 (13.7%)	1.09 (1.00–1.19)
Marital status		
Single	127,296 (8.3%)	0.82 (0.63–1.01)
Married	1,335,263 (87.1%)	2.02 (1.22–2.81)
Divorce or widowed	70,615 (4.6%)	2.97 (2.94–3.00)
Average income < poverty line		
No	1,390,925 (90.7%)	1.71 (1.53–1.89)
Yes	141,749 (9.2%)	4.71 (4.53–4.89)
Possession of occupational injury insurance		
Yes	3,546 (0.2%)	18.20 (18.13–18.27)
No	1,493,331 (97.4%)	42.64 (42.57–42.71)
Geographical location		
Western China	440,360 (28.7%)	1.95 (1.78–2.12)
Central China	538,418 (35.1%)	1.96 (1.79–2.13)
Eastern China	553,295 (36.1%)	1.61 (1.44–1.78)

#### 3.1.2. Socioeconomic Status and OID

Table 3 presents the association between socioeconomic factors and OID. The risk of OID was significantly inversely correlated with age. Males and individuals who lived in rural areas, were illiterate and lived below the poverty line were more likely to have an OID. People without insurance had an OR that was nearly three times higher than individuals with insurance. Additionally, geographical area and occupation were also related to OID in China. 

**Table 3:** Adjusted OR and 95% CI for OID among Chinese workers aged 16–59 years old.

Variable	Reference	Value	OR (95% CI) ^a^
Age	50–59	16–29	0.62 (0.51–0.75) ***
		30–39	0.75 (0.66–0.84) ***
		40–49	0.86 (0.77–0.95) ***
Gender	Female	Male	3.23 (3.21–3.25) ***
Ethnicity	Minority	Han	1.18 (1.17–1.18) ***
Residence	Rural	Urban	1.18 (1.18–1.19) ***
Education	Senior high school or above	Junior high school	3.28 (3.25–3.30) ***
		Elementary	2.46 (2.44–2.48) ***
		Illiteracy	2.55 (2.53–2.58) ***
Marital Status	Single	Married	3.27 (3.24–3.30) ***
		Divorce or Widowed	1.71 (1.69–1.73) ***
Average income < poverty line	No	Yes	1.43 (1.42–1.43) ***
Occupational type	Officers	Research manager or technical personnel	0.66 (0.64–0.67) ***
		Technical personnel in finance	0.63 (0.62–0.64) ***
		Business services	0.49 (0.48–0.50) ***
		Administrative staff	0.75 (0.74–0.75) ***
		Agriculture-related workers	0.96 (0.95–0.98) ***
		Heavy industry workers	1.45 (1.43–1.47) ***
		Light industry and assembling workers	1.12 (1.10–1.13) ***
		Timber workers	1.06 (1.05–1.08) ***
		Production operators	1.05 (1.03–1.06) ***
Possession of occupational injuryinsurance	Yes	No	3.87 (3.83–3.92) ***
Geographical location	Western	Central	0.94(0.92–0.95) ***
		Eastern	0.71 (0.70–0.72) ***

**^a^** OR = Odds Ratio, CI = Confidence Interval; *** means *p* < 0.05.

### 3.2. Discussion

Our study findings were consisted with previous studies [11,14,23,24,25,26,27]. Gender was an important risk factor for OID [13]. Men were employed in high-risk industries more frequently, with a high risk of acquiring an OID [28]. One previous study suggested that construction workers had the highest risk [23]. In the current study, we found that people who engaged in research management, finance and business services had a lower risk of OID compared with timber workers and workers in production operations. Income was another very important risk factor for OID [11,29]. People with a low-income occupation had a higher risk of OID [24]. In the current study, our results suggested that geographical area was related to OID, similar to a previous study [25]. In China, when people are injured in an occupational accident, they prefer to return home, and most live in the Western areas, which are relatively undeveloped and do not offer effective treatment and rehabilitation. This trend is one possible reason that we observed the highest prevalence and an increased OR of OID in the Western area of China. 

Previous studies have found inconsistent associations with OID [28,29]. In Canada, the risk of OID decreased as educational level increased [29], while in Japan, a developed Asian country, the risk of OID increased as educational level rose [28]. China has different conditions than these developed countries. In the current study, the OR of OID for individuals with an elementary school education was lower than for illiterate individuals, and the highest OR occurred among people with a junior high school education. These interesting results might be due to the education pattern in China. In China, the government implemented a “nine-year compulsory education” law, and individuals were not allowed to hold a work position until they had finished their nine-year education. Therefore, illiterate individuals and individuals who only finished elementary school are involved in heavy labor job positions and are therefore at higher risk. 

In the current study, we found the unique result that occupational injury insurance was a significant protective factor for OID. One possible reason contributing to this result is that overall injury insurance coverage is low. At the end of 2007, China had 480 million workers, but only 120 million of them had injury insurance [26], and the proportion decreased for high-risk work positions. For example, in the construction industry, rural migrant workers account for 80% of total workers [27]. At the end of 2006, there were only 50,000 workers with paid injury insurance among 300,000 workers in the construction industry in Tianjin Province [30]. Similar rates were also observed in Hangzhou, Zhejiang Province [31]. Additionally, not all enterprises are required to insure their workers [27]. Therefore, we observed that people without insurance were at higher risk for OID.

The limitations of this study include its cross-sectional design and the lack of a specific cause of the injury such as laceration or collision. Some persons with OID might be institutionalized to receive professional care. Thus, the survey would have excluded these individuals, which may have led to an underestimation of the prevalence of OID. Therefore, the reported research findings indicate a reliable but conservative estimation of the prevalence of OID in China. Health risk factors of OID (*i.e.*, smoking, drinking) were not addressed in the questionnaire, and these factors should be considered in later studies.

## 4. Conclusions 

China’s rapid socioeconomic development depends on the availability of a large number of labor workers. OID leads to a disability burden on individuals, families and health care systems. OID reduction programs, such as creating a safer working environment, providing education and training to workers and greater coverage of occupational injury insurance are necessary to prevent OID and/or improve the lives of people with OID.
